# How to deal with a temporary suspension and restarting your trial: our experiences and lessons learnt

**DOI:** 10.1186/s13063-020-04705-4

**Published:** 2020-09-05

**Authors:** Lynda Constable, Tracey Davidson, Suzanne Breeman, Seonaidh Cotton, Alison McDonald, Samantha Wileman, John Norrie

**Affiliations:** Centre for Healthcare Randomised Trials (CHaRT), Health Services Research Unit (HSRU), University of Aberdeen, Health Sciences Building, Foresterhill, Aberdeen, AB25 2ZD UK

**Keywords:** Temporary suspension, Suspend, Randomised controlled trial, RCT, COVID-19

## Abstract

Whilst the issues around early termination of randomised controlled trials (RCTs) are well documented in the literature, trials can also be temporarily suspended with the real prospect that they may subsequently restart. There is little guidance in the literature as to how to manage such a temporary suspension. In this paper, we describe the temporary suspension of a trial within our clinical trials unit because of concerns over the safety of transvaginal synthetic mesh implants. We also describe the challenges, considerations, and lessons learnt during the suspension that we are now applying in the current COVID-19 pandemic which has led to activities in many RCTs across the world undergoing a temporary suspension.

There were three key phases within the temporary suspension: the decision to suspend, implementation of the suspension, and restarting. Each of these phases presented individual challenges which are discussed within this paper, along with the lessons learnt. There were obvious challenges around recruitment, delivery of the intervention, and follow-up. Additional challenges included communication between stakeholders, evolving risk assessment, updates to trial protocol and associated paperwork, maintaining site engagement, data-analysis, and workload within the trial team and Sponsor organisation.

Based on our experience of managing a temporary suspension, we developed an action plan and guidance (see Additional File 1) for managing a significant trial event, such as a temporary suspension. We have used this document to help us manage the suspension of activities within our portfolio of trials during the current COVID-19 pandemic.

## Background and introduction

The early termination of randomised controlled trials (RCTs) for planned reasons such as statistically based stopping rules, loss of funding, poor recruitment, or futility is well documented [1–6]. However, trials can also be temporarily suspended or put ‘on hold’ rather than terminated, with the real prospect they may subsequently restart.

Several factors can trigger such a suspension: the most common being around a safety concern, or a perceived shift in the risk/benefit balance (or other ethical concerns) [7]. Internal study factors may also play a role, such as the chief investigator or principal investigator being temporarily unavailable, or poor data quality identified during monitoring exercises. External factors may include drug supply issues, trial equipment problems, monitoring inspection findings, or new evidence from other studies. More recently, the global COVID-19 pandemic has also resulted in the temporary suspension of many clinical studies [8].

A search of the literature revealed very little guidance on how to manage a temporary suspension. We are aware of some accounts through ‘word of mouth’ from other trialists. In this paper, we aim to document the experiences and lessons learnt within our UK Clinical Research Collaboration (UKCRC) Clinical Trials Unit (www.ukcrc-ctu.org.uk) (CTU; Centre for Healthcare Randomised Trials (CHaRT), Health Services Research Unit, University of Aberdeen) of a temporary trial suspension.

## Our experience

A publicly funded (UK NIHR Health Technology Assessment Programme) surgical RCT, VUE [9, 10] (Vault or Uterine prolapse surgery Evaluation (ISRCTN86784244)), was temporarily suspended in 2014 in Scottish sites only due to general concerns external to the trial on the safety of transvaginal (TV) synthetic mesh implants.

The trial suspension was triggered when the Scottish Government (June 2014) asked all Scottish NHS Health Boards to consider suspending the use of TV synthetic mesh implants (http://www.parliament.scot/parliamentarybusiness/report.aspx?r=9275&mode=pdf [11]). This was to enable an independent review to consider the ongoing debate on complication rates and underreporting of adverse incidents in the treatment of stress urinary incontinence (SUI) and pelvic organ prolapse (POP) using TV synthetic mesh implants.

VUE incorporates two parallel RCTs evaluating the surgical options for either uterine or vault prolapse which involved or potentially involved the use of TV synthetic mesh implants as part of the surgical intervention (Table 1). In VUE, mesh use was largely dependent on the surgeon’s routine practice, and not specified by the trial. Of note is that the Scottish Government requested suspension was specifically related to the TV use of synthetic mesh; the abdominal use was not included in this suspension.

**Table 1:** The VUE RCTs: two parallel RCTs evaluating the surgical options for upper compartment pelvic organ prolapse

The VUE RCTs [9, 10]	Design and comparisons	Condition	No. of participants recruited at the time of suspension	Mesh in use within the trial	Implications of suspension
**Uterine trial**	Vaginal hysterectomy compared to uterine suspension	Uterine prolapse	224	Uterine suspension procedure dependant on the surgeon’s normal practice (as such, patients may have no mesh, abdominal mesh* or transvaginal mesh)	*Recruitment* Temporary suspension in Scottish sites only (suspending recruitment of new participants and randomisation of consented participants). No change to the rest of the UK. *Intervention* Sites advised to follow local NHS governance on the use of TV synthetic mesh for participants awaiting surgery. This may have deviated from the randomised intervention. *Follow-up* Continued as per protocol
**Vault trial**	Abdominal vault suspension compared to a vaginal vault suspension	Vault prolapse	87	Abdominal procedure requires the use of abdominal mesh*; vaginal vault procedure dependant on the surgeon’s normal practice (as such, patients may have no mesh or may have transvaginal mesh)	

*Abdominal mesh was not included in the requested suspension

VUE was actively recruiting participants and had full approval from an NHS Research Ethics Committee (REC) when the temporary trial suspension occurred. At the time of the suspension in 2014, there was no new emerging evidence within scientific journals or medical literature nor updates or changes to the UK NICE clinical guidelines (IPG215, IPG283, IPG282, IPG284 (no longer available online)).

### Decision to suspend

The Scottish Government’s request to consider suspending the use of TV synthetic mesh in incontinence and POP surgery in Scotland was initially widely interpreted as ‘a ban’ (https://www.independent.co.uk/news/uk/home-news/vaginal-transvaginal-tvt-sling-the-mesh-scandal-nice-guidelines-health-watchdog-nhs-sui-incontinence-a8111721.html [12]), causing confusion not only for the Scottish NHS Health Boards and clinicians but also for patients, trial participants, and our CTU. The trial Sponsor therefore immediately suspended the activity, as described below, in Scottish NHS Health Boards until further information could be reviewed and assessed. The Sponsor and Funder requested urgent information on how the Scottish Government’s request to suspend the use of TV mesh would impact the trial. Trial activity in the rest of the UK did not change as no similar action/request was taken by the UK Government.

### Implementation of the suspension

The temporary suspension involved three Scottish trial sites and incorporated suspending recruitment of any new potential participants, as well as suspending randomisation of eligible participants who had already consented to take part. The randomisation line was closed for the Scottish sites, and for trial participants about to receive surgery (the trial intervention), we advised the local clinical team (principal investigator and research nurses) and local R&Ds to adhere to their local NHS governance decision on the use of TV synthetic mesh (some NHS Scotland Boards did suspend the use of TV mesh, whilst others did not).

The trial team ensured that the key stakeholders (Funder, REC, trial oversight groups: Project management Group (PMG), Trial Steering Committee (TSC), and independent Data Monitoring Committee (iDMC)) together with the local clinical team were notified and kept up to date throughout the suspension period. Emergency trial oversight committee meetings of the iDMC and TSC were organised to discuss the impact or potential impact on the trial, and the ongoing need for robust evidence. The Sponsor also revised their risk assessment.

It is worth noting that the suspension occurred over a relatively short period of time (3 weeks in total) from the initial Scottish Government request (to Scottish Health Boards) to the lifting of the suspension.

### Restarting

Sponsor made the decision to lift the suspension and restart trial activity in Scotland once they were satisfied that all recommendations, questions, or concerns from the key stakeholders had been met. The Sponsor also confirmed their clinical trials insurance continued to cover activity within the trial.

The Scottish sites were made aware of the outcome of the suspension. For these sites, this also involved the need to explain that the lifting of the temporary suspension related only to the research (i.e. the VUE Trial) and not to the Scottish Government’s request to suspend the use of TV synthetic mesh in all SUI and POP surgeries (the Scottish Government had clarified that the use of TV synthetic mesh could continue in clinical trials). This meant that within VUE, the Scottish sites could perform POP surgery using TV synthetic mesh, provided fully informed consent was obtained.

Finally, the randomisation line was reopened on 3 July 2014 for those Scottish sites impacted by the temporary suspension, and the key stakeholders informed that the suspension had been lifted.

## Challenges, considerations, and lessons learnt

An event that triggers a temporary suspension can happen at any phase of the trial (e.g. set-up, recruitment, treatment, or follow-up). Therefore, consideration needs to be given as to who and what is affected by a suspension; be it screening and recruitment (or even contracting and financial contacts in the set-up phase); randomisation; delivery of the intervention or the length, type, or frequency of participant follow-up; scheduled meeting of study oversight committees (TSC and iDMC); progress reports to the funder; and also scheduled monitoring and site visits for quality assurance purposes. Indeed, pretty much any of these trial activities, that constitute a multicentre pragmatic trial, can be adversely influenced by a suspension.

We identified a number of key challenges associated with managing a temporary suspension of unknown duration. These challenges, together with considerations and insights as to how these may be overcome, are described below:

### Planning

The first task identified in managing an event such as a trial suspension is to establish a task force and nominate a ‘Significant Event Lead’. In addition to the Lead, the key team members of the task force included the chief investigator, trial manager, quality assurance manager, and CTU director. Identifying the key stakeholders and creation of distribution lists was also important, as well as prioritising who would require initial contact and standardising the communication outputs.

As this was our first experience of a temporary suspension, we had no formal process to guide us. We therefore created a ‘guidance for significant major events’ document (including delegation of specific actions) as well as an ‘events and actions timeline’ template (Additional File 1) during this period, which was particularly challenging due to the fast-paced nature of the event.

Our guidance detailed the requirements and actions to consider if suspending randomisation, communication/notification of all stakeholders, and the process for responding to press and Freedom of Information (FOI) queries (i.e. who is responsible and who is deputy if that individual is not available). The ‘events and actions timeline’ evolved in real time but was also updated and renewed over time to ensure it was an accurate reflection of what happened, and when.

We highly recommend documenting all the information related to a ‘major event’ (the temporary suspension) in such an ‘events and action timeline’, which could include any immediate corrective/preventive action, and define responsibilities. This timeline document can then evolve over the lifetime of the event and be updated accordingly.

Bringing the task force together following such an event is extremely important to ensure consistency and accuracy in documenting the lessons learnt, as well as updating or making changes to processes (if required) or to ensure preparation for any future similar events.

Maintaining an accurate record of all events is extremely important. We set up a dedicated (and secure) folder to provide a suitable facility for the retention of all relevant documents that staff involved could access. These records could be held electronically, as hardcopy or as a hybrid system. A hard copy or screenshot of any relevant web articles should be retained as these may subsequently become unavailable online over time.

### Communication

Timely, consistent, and accurate communication is key, both within the trial team and to stakeholders. This can be particularly important (as was the case for us) given the potential speed of development of ongoing events and decisions, to ensure everyone could confidently rely on the information given out by the trial office as being correct and authoritative.

The communication needs of all stakeholders had to be met. The stakeholders included the Sponsor, Insurer, Funder, REC, oversight committees, sites, clinical staff, and participants. Careful consideration of what should be shared was necessary. Updates needed to be consistent, and the amount and complexity of detail varied depending on the stakeholder in order not to overwhelm them with too much detail (e.g. what is communicated to trial participants compared to the Sponsor). These communications were coordinated by the ‘Significant Event Lead’.

The mode of communication also required consideration. We used a variety of communication methods to convey the necessary information to all stakeholders such as email, telephone, and meetings as well as use of the site specific (non-public) trial websites (where logins/access could be monitored to verify access to the changing information).

Differing interpretations of the Scottish Government’s request to suspend the use of TV synthetic mesh, as well as media reporting of the event, were particularly challenging, as many Scottish Health Boards interpreted the request differently. For example, it was unclear from the initial report if it was only for incontinence or POP or if it was specifically related to the TV use of mesh in any procedure.

In addition, we realised different stakeholders also had different interpretations of what a temporary suspension actually meant (for example: patients could not be screened; patients could not be recruited; the intervention could not go ahead; there could be no clinics or follow-up), and risk aversion varied amongst these stakeholders.

In order to communicate clearly, we needed to clarify (to ourselves and ultimately to the sites) what a temporary suspension actually meant; in this case, no recruitment or randomisations could go ahead. We prioritised closing the randomisation line ahead of informing sites.

The initial steps in restarting the trial, after lifting the suspension, were primarily to ensure the various stakeholders were happy to continue their involvement. The Sponsor, iDMC, TSC, and Funder needed to be kept fully informed throughout, particularly with any potential impact on the study processes. Agreement from all stakeholders was necessary before the decision to lift the temporary suspension was made.

The media and patient groups can also generate FOI requests (Table 2) to the trial office, Sponsor, REC, Funder, and sites. As a result, the trial office needs to be vigilant to such requests and ensure they are managed and handled according to the Institution’s policy. This ensures such requests are handled by those with the relevant experience and understanding and provided consistency in responses. This can be particularly helpful if the same requests are sent to multiple stakeholders, including the trial office, Sponsor, REC, and Funder.

**Table 2:** FOI requests

The Freedom of Information (FOI) Act 2000 (https://www.gov.uk/make-a-freedom-of-information-request) and The Freedom of Information (Scotland) Act 2002 (FOISA, https://www.gov.scot/about/contact-information/how-to-request-information/) allow public access to information held by public authorities (with guidelines detailing how to respond to these requests).	
This public access to information is actioned through public authorities being obliged to publish certain information on their activities when members of the public request information.	
The public authorities have 20 working days to respond to the FOI/FOISA.	

### Risk assessment

The risk assessment of the trial may need to be reviewed to consider if any changes are required, for example, updates to the safety reporting processes. Uncertainty around the suspension may also result in the risk being reclassified by the Sponsor, potentially involving increased reporting or monitoring requirements.

Our trial underwent an updated risk assessment and was also reclassified by our Sponsor from a moderate to a high risk trial, resulting in increased monitoring and revision of serious adverse event reporting to that in line with drug trials.

### Updates to trial protocol/paperwork

The trial protocol or paperwork may also need revised/amended depending on the reason for a temporary suspension. To make this process as smooth as possible, we recommend any changes to the study protocol or paperwork involve the key stakeholders to ensure everyone has an opportunity to discuss and agree to any required changes.

### Site engagement

The reason(s) and length of a suspension are likely to influence site engagement. For example, if the suspension is due to safety concerns, this could lead to a bigger impact than say drug supply issues.

Our experience taught us that some sites were happy to reopen and recommence all trial activities whilst others were more reserved. Some sites will reopen but not continue to recruit participants due to reticence on the part of the principal investigator (PI)/surgeon and/or the hospital clinical director. Others will open but remain inactive and not recruit any further participants due to a decline in potential and/or willing participants, or sites may remain closed whilst waiting for further guidance/updates, etc.

A temporary trial suspension may also impact potential new sites and sites in set up. Potential sites may then decline to take part in the trial or delay their participation indefinitely.

To maintain engagement of site staff, we prioritised other trial-related activities during the suspension, such as data checks, ongoing training, and regular communication.

Engaging the local research networks to ensure site staff are not moved on to other projects may also be important in retaining the sites’ engagement.

Further training for site staff or trial marketing should be considered if relevant, depending on the reason and length of suspension.

### Recruitment and randomisation

A temporary trial suspension may impact recruitment of trial participants. It may be that some sites recruit more slowly as they either no longer prioritise the trial locally or continue to have ongoing concerns following the suspension.

Once the suspension was lifted, recruitment was closely monitored to establish if it had slowed down. The impact of the suspension and reopening of the trial varied across the three sites; this was related to site and team engagement (as described above). The impact on recruitment on our trial was difficult to evaluate.

The randomisation system we use was designed such that randomisation could be stopped immediately in individual or all sites. When the suspension was lifted, the randomisation line was reopened, and we recommend it is re-tested to ensure there are no problems. There may also be an opportunity to add key information to the randomisation system alerting sites to the suspension, particularly if it is an international trial and time zones make it difficult to communicate with individual sites in a timely manner.

### Delivery of the intervention

For our suspension, we deferred to the local NHS policy for treatment using TV synthetic mesh implants. During this suspension, that meant no participants received their randomised surgical procedure until after the suspension was lifted. Considerations for the delivery of the trial intervention are essential. In non-surgical studies, decisions around the ongoing delivery of the intervention may be more complex—for example, if trial participants are taking study drugs in the community.

### Follow-up

Retention and follow-up of trial participants may also be impacted. Depending on the disease area/trial intervention, one impact of a suspension may be that more frequent participant follow-up is implemented. An increase or decrease on participant questionnaire response rates may also be experienced as heightened awareness may influence whether participants choose to engage. Again, this was difficult to evaluate in our trial.

### Data and analysis

The temporary suspension may have an impact on trial data (amount being collected/integrity/changes prior to the suspension). Consideration should be made to how this will be handled in the data analysis. Differences may be observed in baseline characteristics pre- and post-suspension, and/or there may be an effect on the outcome data. It may be appropriate to do sensitivity analyses to evaluate the data before and after the suspension to reassure the findings are robust. If this is done, it is also important to update the statistics analysis plan (SAP).

### Workload

Not surprisingly, the impact on the trial office will likely involve an increase in workload, possibly resulting in a shift in the types of tasks that require to be undertaken, along with the usual day-to-day work.

This increased workload may stem from addressing participant queries, Sponsor concerns, and changes to study documentation/paperwork and addressing concerns and queries from sites, key stakeholders, and media (through FOI requests, Table 2). In addition, sites may require reassurance that the study will continue and confirmation of new study processes. Some sites may also require extra support to deal with the increase in participant queries.

An unanticipated impact on the trial office workload came from the media and patient groups. This was in the form of increased queries and the need to be more vigilant and consistent in our response.

## Conclusion

Whilst a trial suspension may be short in duration, the impact on the study should not be underestimated. VUE was temporarily suspended for a relatively short period, but the impact continues. Given the recent COVID-19 pandemic, most trials will be experiencing a suspension of some or all of their activities.

In this paper, we have described our experiences of a temporary trial suspension and highlighted the need for further guidance of such a significant trial event. In order to provide some guidance for other trialists who may experience a temporary suspension of their trial, we have detailed the key challenges we experienced, together with insights as to how these may be overcome.

Given our previous experience of a temporary suspension, we were well placed to deal with the suspension of trial activity as a result of the current COVID-19 pandemic. Within our own Unit, 17 trials had recruitment and/or follow-up suspended, or aspects of their intervention delivery or follow-up altered, to accommodate the impact of COVID-19. All these trials successfully used the guidance developed for significant major events and populated the events and actions timeline template (Additional file 1). The actions timeline continues to be helpful for reporting to Funder, Sponsor, REC, oversight committees, participants, sites, clinical staff, and within our immediate trial teams. It is also likely to prove helpful when reporting trial results in the future.

As a Unit, we have developed and tested our significant major events and timeline documents in two very different scenarios and plan to continue to use them for any future events.

## Supplementary information

**Additional file 1.**

## Data Availability

Not applicable
