# Supplementary material for: How to deal with a temporary suspension and restarting your trial: our experiences and lessons learnt
Source: Trials. 2020 Sep 5;21:765. doi: 10.1186/s13063-020-04705-4 (PMC7474317; doi:10.1186/s13063-020-04705-4)
Supplement: Supplementary file 1 — Additional file 1. [file 13063_2020_4705_MOESM1_ESM.pdf]

GUIDANCE FOR SIGNIFICANT MAJOR STUDY EVENTS
(e.g. trial suspension for safety reasons)

CHaRT
Centre for Healthcare Randomised Trials

Once the event has been has been confirmed as 'significant' by the CHaRT Director or senior management group, please use the guidance below to help manage the event.

The event should be fully described in a detailed timeline, using the 'Events and Actions Timeline' template.

Communications

Nominate a 'lead'	Director/Senior TM/TM/ CI (or delegate)
Nominate a Task Force	Director, Senior TM, Senior IT, QA manager, TM, CI<br>(or delegate).<br>*At least 2 senior members of CHaRT*
Identify key stakeholders (who should be initially contacted & informed of Event (e.g. suspension))	1. Sponsor (check T&C of Insurance and relevant Sponsor SOP(s))<br>2. Funder<br>3. PMG<br>4. MHRA (if applicable)<br>5. REC<br>6. R&D<br>7. DMC/TSC (and review stopping rules in charter)<br>8. Trial sites<br>9. Participants<br>10. UoA Communications Office
Create 'appropriate' distribution lists for communications	Immediate Trial Office team (CI/TM/DC)<br>+ PMG<br>+ DMC/TSC<br>+ full stakeholder list<br>etc.
Standardise and agree communication outputs	E.g. emails to sites etc.

Requirements and Actions to consider

Action	Task	Responsible person(s)	Response time/time frame
	The 'lead' needs to convene a meeting with the Task Force to generate the plan of action as soon as practically possible.	Lead	Ideally within 24hr
**Understand 'Event'**	Understand what the event is and what the implications might be. Ensure transparency in how this event is to be communicated.	Task Force	
**Create a timeline**	Use the 'Events and Actions Timeline' template to collate a timeline detailing all the issues relating to the event and the immediate corrective/preventive action taken, where appropriate	Task Force	Ongoing

Action	Task	Responsible person(s)	Response time/time frame
**File the Events and Action Timeline**	File the 'Events and Actions Timeline' in an appropriate e-folder so the 'Task Force' are able to access and edit the document. Access may need to be limited/restricted	Task Force	Ongoing
**Define responsibilities of the CI and CHaRT Senior Management / trial team**	Note: Consideration if CI is unavailable – nominate (and document) who has been delegated responsibility	CI / TM	Ongoing
**Arrange meeting / teleconference with Sponsor to confirm requirements**	Review relevant Sponsor procedures and timelines	CI / TM	Ongoing
**Consideration if recruiting: randomisation service**	Suspend randomisation service (define which sites) and consider adding a message to the service to alert sites of the suspension<br><br>Switch randomisation service back on	Senior programmer & CI/TM	Start<br><br>End
**Considerations for unblinding**	Consider if participants/ study team(s) are required to be unblinded?	Trial team/senior programmer/statistician	
**Notify sites**	1. Initial contact and consider engaging with local support research network groups<br>2. Consider which trial activities need prioritising<br>3. Follow-up information explaining 'Event' in more detail (consider setting up a specific folder/area on the trial website for such communications)<br>4. Confirmation of final outcome/decision<br>5. Consider if further training is required	Trial team	
**Notify participants**	Do they need to be made aware of new information?<br><br>Does the PIL need updating (may be stipulated by the DMC / TSC / Sponsor)?	Trial team	
**Notify REC**	Initial Contact (consider if this is a substantial amendment, they may inform if substantial/non substantial)<br><br>Confirmation of final outcome / decision	Trial team	
**Notify MHRA (if appropriate)**	May need to communicate with the MHRA	Trial team	

Action	Task	Responsible person(s)	Response time/time frame
**Notify TSC &/or DMC**	May be required to arrange TC or meetings to discuss (& respond to) issues relating to 'Event'	Trial team	
**Review/update/draft risk assessment**	May need to consider changes to safety reporting, monitoring, site visit, frequency of TSCs/DMCs etc.	Sponsor	
**Review study guidance / local SOPs**	May need to revise / update local study guidance / local SOPs	Trial team	
**Review Trial protocol / paperwork**	May need to revise / update protocol / paperwork with input from appropriate stakeholders (may depend on outcome of the risk assessment)	Trial team	
**Inform REC & Sponsor**	If updates to protocol and study paperwork made then approval will need to be sought from REC & Sponsor	Trial team	
**Consider impact on trial data**	May need to review how the event will impact the data analysis. May need to revise SAP (consider if protocol update required and inform REC)	Trial team and statistician	
**Consider if confirmation is required from any of the stakeholders prior to, e.g. lifting suspension at sites/ randomisation service (*may be required prior to notification to sites of final outcome/ decision*)**	1. Sponsor/Research Governance<br>2. Funder<br>3. MHRA (if applicable)<br>4. REC<br>5. R&D<br>6. PMG<br>7. DMC/TSC	Task force	
**If contacted by press (Publicity)**	Refer any issues to the Director/ Task force/Sponsor/Funder/UoA Communication Office as appropriate	Task force	
**If contacted regarding Freedom of Information (FOI)**	Refer any issues to the Director/ Task Force/Sponsor/Funder/UoA FOI office for trials sponsored locally or the equivalent if sponsored elsewhere	Task force	
**Sign off**	Following completion of the event ensure it is formally logged on the 'Events and Actions Timeline' as being signed off by the nominated lead		

EVENTS AND ACTIONS TIMELINE

Centre for Healthcare Randomised Trials

<<Trial name>> – <<Brief description of event>>

This is a summary of the action taken following …<<please detail>>.

DATE	ISSUE (*name trial if multiple trials involved*)	IMMEDIATE (CORRECTIVE) ACTION TAKEN BY CHaRT	ACTION COMPLETED BY	COMMENTS/RELEVANT DOCUMENTS/LINKS*	SUBSEQUENT ACTION/ OTHER COMMENTS

Please add rows as required

**Please be aware that web links may break over time, so if the information being provided is important it might be best to save and keep a copy of the link or print out*

EVENTS AND ACTIONS TIMELINE

To be signed by the named Lead

I confirm that this ‘Event’ is complete and can be signed off

Name: (PRINT) ______________________________

Signature: ______________________________

Date:

D	D	M	M	Y	Y	Y	Y
